# 

**Published:** 1801-06

**Authors:** 


### *Advertisement to the Medical World.*

THE Proprietors of the periodical Work, entitled THE LONDON MEDICAL REVIEW and MAGAZINE, having determined in future to confine the object of that work *ſolely* to a full and comprehenſive Analyſis of NEW MEDICAL BOOKS, domeſtic and foreign; and having agreed to recommend their friendly Correſpondents to tranſmit their future communications to the MEDICAL and PHYSICAL JOURNAL, the Editors of the latter work announce this new and improved arrangement with much ſatisfaction, and earneſtly invite the Correſpondents of the London Medical Review and Magazine to honour them with their communications; aſſuring them, that they will on all occaſions be treated with due reſpect and diſtinction.

The Editors of the MEDICAL and PHYSICAL JOURNAL take this opportunity to repeat their acknowledgements to the medical world, for the continued and increaſed ſupport with with which their exertions have been attended; and as THE MEDICAL and PHYSICAL JOURNAL will continue to be carried on *precisely upon the same plan, and with the same spirit as heretofore,* the Editors venture to promife their readers a confiderable addition to the value of this work, from the acquifition of the correfpondence which has hitherto been peculiar to the LONDON MEDICAL REVIEW and MAGAZINE.

This laft mentioned work will in future be publifhed as ufual, on the firft day of every month, under the title of the LONDON MEDICAL REVIEW, and will recommend itfelf to a continuance of the patronage it has been honoured with, by the copioufnefs and the independence of its criticifms on every new publication which is connected with the ftudies or purfuits of medical men. The plan of this work being, therefore, wholly different from that of the Medical and Phyfical Journal, the profeffional reader will be able to indulge his peculiar views in the purchafe of a periodical medical work, at half the previous expence. Thofe who are in queft of new facts, obfervations, experiments, and cafes, will confult the MEDICAL and PHYSICAL JOURNAL, in which they will alfo meet with a Retrofpect neceffarily brief, of all the novelties in medical literature; and thofe who would enter more at large into the critical difcuffion and hiftory of the practice of medicine, and its collateral fciences, will probably feel a ftrong intereft in the perufal of the LONDON MEDICAL REVIEW. Many gentlemen, who, not regarding the expence, determine to gratify themfelves by reading both works, will hav the advantage, in confequence of this arrangement, of not purchafing the fame intelligence twice over. In every point of view, the Editors of both works confider the arrangement they have adopted as matter of congratulation to their friends and correfpondents, and they hope the public at large will be of the fame opinion,

*London, April* 25, 1801.

### INDEX.

Page.

ABILDGAARD, Prof. on injecting medicines into the veins of horses 399
Abscess in the groin, Bradley's case of 219
Acid concrete, Trotter Dr. on 75
Acids, Beddoes Dr. on the anti-veneral, power of 72
Action febrile, Dr. Mossman's treatise on, announced 400
Adams, his case of pins swallowed 245
——— Dr. on pulmonary consumption 307
Advertisement to the medical world 591
Addington on cow pox, review of 292
Advertisement to the medical world 495
Affusion cold in fever, Dr. Garnet on 12
——————— Dr. Trotter on 75
Aikin, C. R. on the cow pox reviewed 383
Alkalis, Stutz, Dr. on the medical use of 470
Amputated limbs, Pott's on the means of supplying 277
Analysis chemical of vegetables, Hermbstæds Dr. 361—467
Angina maligna, Headly on 424
Animal pregnation, Pulley's essay on the proximate cause of, announced 400
Antimony, Cit. Vaquelin's discovery of siliceous earths in 199
Apoplexia Hydrocephalica, Dr. Garnett's case of, with remarks 121
Arcanum Mathieus against the tapeworm 398
Arsenic in Cancers, Simmons on 251
Artery radial, Yelloly, Dr. on it 282
Arthritic complaints, Girault on warm muriatic baths in 587
Arthritic complaints, Brefeld on the use of oils in 490
Artillery hospital, review of Dr. Rollo's account of it 383
Asthenology, Dr. Struve's review of 377
Asclepiades, Dr. Burdach's reviewed 373
Batty, Dr. letter to, on Hull's essay on phlegmatia dolens, by Dr. Ferriar 5
——— Dr. letter to him from Dr. Cappe on the Cow pox 25
———— his lectures announced 493
Baudeloque's researches, Dr. Hull's translation of, announced 493

Page.

Beddoes, Dr. on the anti-venereal power of acids 73
Bellamy's case of fracture of the cranium 255
Bell, C. his system of dissections 579
* Baeta, Dr. his comparative view of the theories, review of 373
Beer, Dr. on a remarkable degeneration of both kidnies 303
Bevan on vaccine inoculation 455
Blair, his work on medical and operative surgery announced 200
Blaine, Dr. his communication of Cainsh's cases of plague 538
BOOKS, MEDICAL, CRITICAL RETROSPECT OF
Dr. Hull on phlegmatia dolens 92
Gmellin's history of chemistry, Dr. Struve's triumph of medicine, 175 Prof. Grens elements of chemistry, *ib.* Fodere's treatise on the wen, cretinisn, and influence of foul air on human understanding, 175. Cit. Lacretelle's natural history of salamanders, 177. Prof. Reich's theory and practice of fever, 178. Chisholm's, Dr. essay on malignant, pestilential fevers, 184. Prof. Walter on diseases of the kidnies and bladder, 285. Philosophy of medicine, 286. Parkinson's chemical pocket book, 287. Whyte's observations on gout and rheumatism, 288. Dr. Murray's remarks on the situation of the poor in the metropolis, 289. Dr. Rush on the yellow fever, lectures on animal life, and observations on the origin of the yellow fever in Philadelphia, 291. Dr. Maclean on the Plague, 292. Addington on cow-pox, 292. Dr. Barry on cow-pox, 293. Dr. Willichs on the physical education of children, 293. Dr. Schelver's elementary doctrine of organic nature, 369. Prof. Swartz's Swedish Flora. Dr. Juch on coffee and its substitutes, 371. Dr. Hedwig on ferns, 372. Dr. Burdach's Asclepiades, 373. C. Bells system of dissections. Dr. Struves asthenology, 377. Whateley on the gonorrhea virulenta in men, 379. Dr. Rollo's account of the Royal Artillery Hospital, 383. C. R. Aikin on the cow-pox, *ib.* Dr. M'Donald on the vaccine inoculation, 384. Mr. Creaser on, *ib.* Dr.

Page

Odier, on do. 386. Dr. Colon on do. 386. Dr. Baeta's comparative view of the theories and practice of Dr. Cullen, Brown, and Darwin, 388. Doctor Frank's instruction for the knowledge and choice of a physician, 475. Prof. Bichat's reflections on life and Death, 476. Whately on strictures in the urethra, 479. Dr. Hooper's medical dictionary, 482. Dr. Willan's reports on diseases in London, *ib.* Chevaliers introduction to a course of lectures, 486. Dr. Hogarth's letter to Dr. Percival, on the prevention of infective fevers, and on the prevention of the American pestilence, 582. Instruction relative to self-preservation during the prevalence of contagious diseases, 582.

Baeta, Dr. his comparative view of the theories and practice of Drs. Cullen, Brown, and Darwin, in fever and rheumatism 388

Barks medicinal, Maton, Dr. on 33

Barytes muriated, Durr, Dr. on the solution of 303

Barlow on premature delivery 40

Bartley on artificial musk in hooping cough 137

——— on digitalis 250

Bell, review of his system of dissection 373

Bichat's, Prof. review of his researches on life and death 476, 563

Bichat's, Prof. review of his physiological researches on life and death 563

Bladder and kidnies, diseases in, review of Prof. Walter on 284

Blindness from suppressed catamenia cured by electricity, case of, by Mr. Ricards of Brentford 68

Borrett's case of crural hernia 113

Boeckmann on the illumination of rotten wood

Bones, preternatural growth of, Cit. Saucerotte's case of 402

Bostock, Dr. his account of diseases in the Liverpool Dispensary 117

Boswell's Case of vaccine 341

Boucher, Cit. his observations on elm trees 492

Bronchocele, King on 38—355

Bradley, Mr. his cases in surgery communicated by Mr. Pearson 219—321

——— his case of abscess in the groin 219

——— Tertian intermittent, &c. 227

——— Dr. letter from Dr. Stokes relative to cautions respecting the inoculation of cow pox 17

——— Reed's letter to, on Man's artificial leg 493

Page

Bradley, on yeast in contagious fever 145

Branson's letter to Dr. Pearson, on the insusceptibility of vaccine contagion in persons having previously had the small-pox 160

Brefeld on the use of oils in arthritic complaints 490

——— cold water in dysentry 491

Brera, Prof. on the internal use of phosphorus 396

Bristol dispensary state of diseases in 317—411—509

Bristol Dispensary, state of diseases in 317 411

——— Infirmary, account of diseases in 411

Bronchocele, Fordere's treatise on, reviewed 176

Brown, Cullen, and Darwin, Drs. theory and practice of fever and rheumatism, comparative view of 388, 577

Burdach, Dr. review of his Asclepiades 373

Burns, Wawn on 55

Burns and scalds, Navalis, jun. on 236

Caloric Hermbstaedts, new theory of 199

Cameron on tincture of ladycow as a substitute for opium 546

Cancer, Dr. Nisbet's reply to Oliphant's information on Mrs. Crab's case of 76

——— Simmons on arsenic in 251

Cappe, Dr. on the cow-pox 25—383

——— in reply to Mr. Franks 31

Carlisle, his plan for a convalescent establishment 305

Carpue, his lectures announced 493

Cartwright on an improved mode of applying caustic to strictures in the urethra 513

Cardialgia, Swift's case of 519

Caustic, Cartwright on, an improved method of applying it to strictures of the urethra 513

Cæsarian operation, Dr. Kurzwig's cases of

Children, new born, Prof. Hufeland on the Erysepelas in 511

Chemistry, Prof. Gmellin's history of, reviewed 173

——— elements of Prof. Gren's, reviewed 175

Children, Struve, Dr. on the physical education of, reviewed 293

Chevalier's introduction to a course of lectures, review of 486

Chisholm, Dr. his essay on malignant pestilential fever reviewed 184

Clitoris, case of an extraordinary enlargement, of *(with plate)* by Simmons 1

Page

Contagion variolous, Franks on
Convalescent establishment, Carlisle's plan for one 305
Corradori on the illumination of rotten wood 583
Corse's discoveries relative to the coupling of elephants, and their time of gestation 588
Coffee, and its substitutes, review of Dr. Juch on 371
Collier on the use of zincum vitriolatum against poison by laudanum 165
Colon, Dr. on vaccine inoculation, review of 386
Consumption pulmonary, Adams, Dr. on 307
Corradori, Dr. on Tremella Nostoc 397
——— his observations on glow-worms 397
Correspondents to 104, 200, 304, 400, 495
Couper, Dr. on generation 404
Cow-pox, cautions respecting the inoculation of, by Dr. Stokes 17
——— Lubbock, Dr. on 23
——— Cappe, Dr. on 25—338
——— Sharpe on 111
——— Hill on 152
——— Woodforde, Dr. on 152
——— Marshall, Dr. on 263
——— King on 281
——— Addington on, review of 292
——— Barry, Dr. review of 293
——— C. R. Aikin on, review of 383
——— new edition of Dr. Jenner on, announced 400
Craib, Mrs. Dr. Nisbet's reply to Mr. Oliphant's information on her case of cancer 76
Cranium, Bellamy's case of fracture of 255
Creaser, Dr. on vaccine inoculation, review of 384
Cretinism, Fodere's treatise on, reviewed 175
Critical Retrospect of Medical Literature, v. *Books Medical*, *Critical Retrospect of*,
Croup, cases of, communicated by Dr. Maton 516
Cullen, Brown, and Darwin, Drs. comparative view of their theories and practice in fever and rheumatism 388
Custance on yeast 143
Darwin, Cullen, and Brown, Drs. comparative view of their theories and practice in fever and rheumatism 388, 577
Davis's case of incipient mortification 534
De Carro, Prof. on vaccine inoculation 175
De Carro, Prof. his letter to Dr. Jenner 351
Delivery, premature, Barlow on 40
——— Headly on. 312
Death and life, Prof. Bichat's physiological researches on, reviewed 563
Dentition, difficult, Dr. Wichmann's theory of, reviewed 567
Diabetes, Lubbock, Dr. on 57
Dictionary, medical, Dr. Hooper's, review of 482
Diet, Nisbett's, Dr. his systematic work on, announced 304
Digitalis purpurea, Magennis, Dr. on its effects in curing phthisis pulmonalis 202
——— Bartley on 259
——— Patterson, Dr. on the use of 441
Diseases in the Bristol dispensary, state of 317, 411, 509
——— from 1st March to 1st April 411
Diseases in the Finsbury dispensary, monthly report of, from 20th November to December 20
——— from 20th December to January 20 104
——— from 20th January to February 20 298
——— from 20th February to March 20 97
——— from 20th March to April 20 413
Diseases in the Liverpool Dispensary between Jan. 1, 1800, and Jan. 1, 1801 118
Diseases in an eastern district of London, from 20th January to 20th February, 1801 301
Diseases in London, Dr. Willan's report of, reviewed 482
——— in an eastern district of London, from the 20th November to 20th December, 1800 197
——— 20th December to 20th January, 1801
——— 20th January to 20th February 301
——— 20th February to 20th March 319
——— 20th March to 20th April 411
——— 20th April to 20th May
Diseases in the Westminster hospital, from 22d October to 26th November 16
——— 24th December to 25th March 329
——— 25th March to 27th April 416

Page.
Dislocations, Evans's cure of 521
———— and casualties for the year 1800 302
Dissections, C. Bell's system of, reviewed 373
Distortion of the feet, Sheldrake on the cure of 9
———— Watt on 426
Dropsy, Swift's case of 348
———— Rolfe's cases of 405
Dr. Dennison and Dr. Squires on the theory and practice of midwifery, and the diseases of women and children 591
Dunning on vaccine inoculation 155
Durr, Dr. on the solution of muriated barytes 303
———— on sudor pedum 303
———— on the head-ach 397
Dysentery, Brefeld on cold water in 491
Dyspnœa, Bartley on artificial musk in the cure of 137
Earth, Silicious, Cit. Vaquelin's discovery of in antimony 199
Edinburgh, account of the medical school at 523
Edlin on yeaft 422
Edwards on ulcers 417
Electricity, blindness, from suppressed catamenia, cured by it, by Mr. Ricards 68
Elephants, Corse's discoveries relative to their mode of coupling and time of gestation
Elm trees, Cit. Boucher's observations on 492
Encyclopædia, new Medical, announced 493
Epilepsy, Hodgson's case of 347
———— Kinglake, Dr. on 435
Erysipelas of new-born, Prof. Hufeland on 511
Essays, medical, Oxley's 329
Evan's, his case of dislocation 521
Eye, case of a disease of it, by Humphreys 241
Eye, retina of, Wantzel's observations on Herne's account of an orifice in it 585
Falconer, Dr. on the Portland powder 417
Febrile action, Dr. Mossman's treatise on, announced 400
Feet, distortions of, Watt on 426
———— Sheldrake on 456
Ferguson, Dr. of Aberdeen, his essay on fever, announced 200
———— on the tongue, and its diseases 247
Fermer, letter to him from Mr. Sharp on the cow pox 113
Fever, contagious, among the poor, observations on the prevention of 589

Page.
Ferns review of Dr. Hedwig on 372
Fever, Mossman, Dr. on 129
———— Prof. Reich's theory and practice of, reviewed 178
———— Malignant pestilential, review of, Dr. Chisholm's essay on 184
———— and rheumatism, comparative view of, Drs. Cullen, Brown, and Darwin on 388, 577
Fielding on hooping cough 141
Finsbury dispensary, monthly report of diseases in, from Nov. 20 to Dec. 20 97
———— Jan. 20 to Feb. 20
———— Feb. 20 to March 20
Flora, Swedish, Prof. Swartz's reviewed 370
Fodere, review of his treatise on the wen, cretinism and influence of moist air on human understanding 175
Forbes, Charles, Esq. his letter to Dr. Kier on the anti-venereal power of acids 73
Fracture of the cranium, Bellamy's case of 255
Franks, Mr. Dr. Cappe in reply to 31
———— on variolous contagion 81—146
———— Dr. review of his instruction for the knowledge and choice of a physician 475
Gaertner, Dr. on the illumination of rotten wood 583
Garnett, Dr. on cold affusion 12
———— his case of apoplexia hydrocephalica 121
Generation, Conyer, Dr. on 404
Girault on warm muriatic baths in arthretic complaints 587
Glow worms, Corradori, Dr. on 397
Gmellin, Prof. review of his history of chemistry 173
Golis, Dr. on the blue fever 586
Gonorrhea, virulenta, in men, Whately on, reviewed 379
Gout and rheumatism, Whyte on, review of 288
Gren, Prof. review of his elements of chemistry 175
Groin, case of abscess in, by Bradley 219
Grose on yeast, in reply to Custance 270
———— vaccine innoculation 430
Hardman on premature labour 253
———— on hysterotomy 353
Harle, Prof. his case of morbus maculosus hæmorrhagicus 489
Harrison, Dr. on vaccine inoculation 108
Hæmorrhagicus maculosus morbus, Prof. Harle's case of 489
Hebb's case of small pox 536
Headach, Durr, Dr. on 397
Headly on premature delivery 312

Page.
Headly on angina maligna 424
Hedwig, Dr. on Ferns, reviewed 372
Henry, W. of Manchester, his work on chemistry, announced 200
Hertostaed's new theory of caloric and certain acids 199
——— chemical analysis of vegetables 361, 467
Hernia Spry's, case of 105
——— Crural Borret's, case of 113
Hill on cow pox 151
Hooper, Dr. review of his medical dictionary 482
Hooping cough, Fielding on 143
Horses, Prof. Abildgaard on injecting medicines into the veins of 399
Houseleek, Cit. Vauquelin's mode of extracting malat of lime from 198
Hull, Dr. on phlegmatia dolens 93
——— his answer to Dr Ferriar 493
——— his translation of Baudeloques researches, answered 493
Hufeland Prof. on the Erysipelas of new born children 511
Hutchinson on vaccine 359
Humpage his case of tumefied labium 53
Humphryes his case of a disease of the eye 241
Hydrocephalus, Ricards on 342
Hysteria, M'Laren's case of 235
Hysterotomy, Hardman on 353
Ice vinegar, Prof. Lowitz's new mode of preparing 395
Inoculation, vaccine
——— Woodville, Dr. on
——— Peirson, Dr. on
Inoculation, vaccine, Dr. Jenner on the orign of
Inoculation, vaccine, at Paris, report of the committee on 99
——— signatures added to the testimonial in favour of 103
——— Harrison, Dr. on 108
——— Dunning on, 155
——— De Carro, Prof. on 157
——— Maddock on 161
——— Marshall, Dr. on 315
——— letter to the mayors of Paris on 356
——— M'Donald, Dr. on review of 384
——— Creaser, Dr. on, *ib.*
——— Odier, Dr. on review of 386
——— Colon, Dr. on review of 386
——— Watt on 430
——— Gren on 452
——— Bevan on 455
Jaques's, Dr. cure of poison 542

Page.
Jaw locked, Bradley's cure of 321
Jenner, Dr. letter to, from Dr. De Carro 351
——— new editions of, on the cow-pox announced 400
——— the rev. Mr. on vaccine inoculation 401
——— naval medical officers, address to 432
Jennerian medal the, Dunning on 155
——— naval subscribers to 434
——— on the 74
Jennerian Inoculation, Trotter, Dr. on
——— Marshall, Dr. on 316
Juch, Dr. on coffee and its substitutes 371
Kidnies and bladder diseases in, review of, Prof. Walter on 284
——— both remarkable regeneration of, Dr. Beer on 303
Kinglake, Dr. on Epilepsy 435
Kurzwig, Dr. of Riga, his cases of Cæsarian operations 584
Kynion on swelled legs 242
Labour, premature, Hardman on 253
Lacretelle's, Cit. natural history of salamanders reviewed 177
Lamb's case of white swelling 346
Larynx divided, Milburn's case 346
LECTURES ANNOUNCED,
Bradley, Dr. on the theory and practice of medicine, 103. Batty, Dr. on the theory and practice of midwifery, and diseases of women and children, *ib.* Pearson, Mr. on the principles and practice of surgery, *ib.* Mr. H. Leigh Thomas on practical surgery, *ib.* Mr. Wilson on anatomy, physiology, pathology, and surgery, *ib.* Mr. Chevalier on the principles and practice of surgery, *ib.* Mr. Stancliffe on chemistry. Dr. Pearson on the practice of physic, materia medica, and Chemistry, 200. Dr. Batty's, 304. Macartney's, *ib.* Fox's, *ib.*
Labium tumefied, Mr. Humpages case of 53
Lassus, Cit. on the deleterious effects of opium 491
Lassus, Cit. on the deleterious effects of opium 491
Leg artificial Man's, Reed's letter to Dr. Bradley on 493
Legs, swelled, Kynion on 242
Lehnhardt's, Dr. salubrious liquor for pregnant women 399
Lewin, Dr. on the use of artificial musk 544
Life and death, Prof. Bichat's researches on, reviewed 476

Page.
Limbs, amputated, on the means of supplying the loss of 277
Liverpool Dispensary, account of diseases in from Jan. 1st, 1800, to Jan. 1st. 1801 118
Life and death, Prof. Bichat's physiological researches on, reviewed 563
Lime, malat of, Cit. Vauquelin's mode of extracting it from houseleek 198
Lime tree, Ventenat on, its genius 394
Lime tree, Ventenat on the genus of 394
Literature, medical and physical, critical retrospect of 92—475
London, Murray, D. on the situation of the poor in, reviewed 289
London, state of diseases in 194
——— review of Dr. William's reports of diseases in 482
Lowitz, Prof. his new mode of preparing ice vinegar. 395
Lubbock, Dr. on cow-pox 23
——— on diabetes 56
——— ——— his letter to Dr. Batty, correcting an erratum 164
M'Donald, Dr. on vaccine inoculation, reviewed 384
Man's artificial leg, Reed's letter to Dr. Bradley on 493
Maton, Dr. communications on cases of crocys, by 516
Maddock on vaccine inoculation 161
Magennis, Dr. on digitalis, in the cure of phthsis pulmonalis 201
Mamma inflamed, Wagstaff's case of 239
Marshall, Dr. on cow pox
——— on the jennerian inoculation 315
Mathieu's arcanum, against the tape-worm 308
Maton, Dr. on medicinal barks 33
Mayor's of Paris, letter to, on the vaccine inoculation 356
M'Larens case of Hysteria, cured by musk 235
Medical prize questions for 1801 588
Medical Dictionary, Dr. Hooper's, viewed 482
Medical Society, list of officers and committees of 314
Medical and physical intelligence 303 394—489
Medical and physical literature, foreign and domestic, critical retrospect of 173—284—369
Medal, Jennerian, Dunning on it 154
Medical essays, Oxleys 329
Medical prize questions for 1781 313
——— school at Edinburgh, account of 524
Medical and physical intelligence 99-198
Medical and physical literature, critical retrospect of 563

Page.
Memoranda chemica, Parkinson's, review of 287
Mezerium, Roger's, Dr. on 278
Milburne's case of divided larynx 346
Monstrosity, Syer on 170
Mortality, general bill of, for the year 1800 302
Moore's case of Synocha 232
Mossman, Dr. on fever
——— ——— his dissertation on febrile announced 400
Murray, Dr. on the situation of the poor in London reviewed 289
Musk, artificial, Bartley on, its use dyspnœa 137
——— its use in the cure of hysteria 235
——— Lewin, Dr. on the use of 544
Navalis, jun. on burns and scalds 236
Naval medical officers, address to, Dr. Jenner 432
———subscribers to the jennerian medal 434
Navy medical officers gold
Nemnich, Dr. of Hamburg, his Nosological Dictionary announced 58[illegible]
Nisbet, Dr. his reply to Mr. Oliphant's information on Mrs. Craib's case of cancer 76
——— his systematic work on diet announced 304
Odier, Dr. on vaccine inoculation, review of 389
Oils, Brefeld, on the use of in arthritic complaints 490
Oliphant's information on Mrs. Craib's case of cancer, Dr. Nisbet's reply to 76
——— further information on Mrs. Craib's case of cancer, with additional cases 183
Opium, Lassus, Cit. on the deleterious effects of 491
——— tincture of lady-cow, as a substitute for, Cameron on
Opium, Cameron on tincture of lady-cow, as a substitute for 546
Organic nature, review of Dr. Schelver's elementary, doctrine of 368
Ossicula auditus, observations on them 349
Ovarium of Women, Prof. Roose on the corpora lutea in 587
Oxley's medical essays 329
Patterson on the use of Digitalis 441
Pearson, Dr. on the inoculated vaccina 87
Pedum sudor, Durr, Dr. on 303
Philadelphia, Rush, Dr. on the malignant yellow fever, of, reviewed 291
Phillips on vaccina 547
Phlegmatia dolens, Ferriar, Dr. on, Hull's essay on 5

Page.
Phlegmatic dolens, Hull, Dr. critical Retrospect of his essay on 93
Phosphorus, Brera, Prof. on the internal use of 396
Phthisis pulmonalis, Magennis, Dr. on the effects of digitalis in the cure of 201
Physician, Dr. Franks's instruction for the knowledge and choice of one reviewed 475
Physiological researches on life and death, Prof. Bichat's, reviewed 563
Physiological researches on life and death, Prof. Bichat's, reviewed 563
Pins swallowed, case of, by Adams 245
——— Whyte on 245
——— Maclean, Dr. on, review of 292
Plague, true, Tainch's cases of 538
Pneumalkali described by Dr. Harhnemann, experiments, on by Prof. Tromsdorf and Klaproth
Poison, Jaques, Dr. his case on 542
Pole's case of vaccine 340
Poor in London, Dr. Murray on the situation of, reviewed 289
Portland powder, Dr. Falconer on 417
Potts on the means of supplying amputated limbs 277
Pox, small, Purton's case of 403
Pregnation, animal, Pulley on the proximate causes of announced 400
Prevention of contagious fever among the poor, observations on 591
——— Hebb's case of 536
Prize questions, medical, for 1801 313
Pulley, his essay on the proximate cause of animal pregnation announced 400
Purton, his case of small-pox 403
Pulmonary consumption, Adams, Dr. on 307
Questions, prize, medical, for 1801 313
Rees, Dr. his lectures announced 493
Reich, Prof. review of his theory and practice of fever 178
Reid, his letter to Dr. Bradley on man's artificial leg 493
Retina of the eye, observations on an orifice in the 585
Rheumatism and fever, Dr. Baeta's comparative view of the theories and practice of Drs. Cullen, Brown and Darwin 388
Ricards on hydrocephalus 342
Ring on brenchocele 38—355
——— on the cow-pox 281
——— on vaccine 354
Rogers, Dr. on mezereum 278
Roose, Prof. on the corpora lutea in the ovarium of Women 587

Page.
Rolfe's cases of dropsy 405
Rollo, Dr. review of his account of the Royal Artillery Hospital 383
Salamanders of France, Cit. Lacretelle's history of, reviewed 177
Saucerotte's case of preternatural growth of the bones 492
Scabie's account of a new ointment for
Sharp on cow-pox
Shearsmith's case of incysted tumour 520
Sheldrake on the cure of distortion 9—456
——— in reply to Watt 264
Simmons, R. his case of an extraordinary enlargement of the clitoris (*with plate*) 1
——— on vaccine 134
——— in reply to Syer 254
——— on arsenic in cancer 251
Small-pox, Purton's case of 403
Small-pox, Hebb's case of 539
Society, medical, list of officers and committees of the 314
Spallanzani on the illumination of rotten wood 583
Spry, his case of hernia (*with plate*) 105
——— his letter recommending a systematic arrangement of diseases 107
Stokes, Dr. his letter to Dr. Bradley on the cautions respecting the inoculation of cow-pox 17
Strictures in the urethra, Whately, Dr. on, reviewed 479
Struve's triumph of medicine reviewed 175
Stutz, Dr. on the medical use of alkalis
Strictures in the urethra, Cartwright on an improved method of applying caustic to 518
Stutz, Dr. on the medical use of alkalis 470
Surgery, Bradley's cases in, communicated by Pearson 219
Swift's case of dropsy 348
——— cafes of an eruption occasioned by Jesuit's drops and cardialgia 519
Swartz, Prof. his Swedish Flora reviewed 379
Syer on monstrosity 170
——— Simmons in reply to 254
Synocha, Moore's case of 232
Tainsh's cases of plague 538
Tape-worm, Mathieu's arcanum against 398
Tartar Emetic, Vanquelin's mode of preparing.
Tertian intermittent, Bradley's case of 226
Thomson, Dr. his work announced 493
Thilo, Dr. his galvanic experiments with salt-petre 587

Page.
Tongue and its diseases, Ferguson, Dr. on 247
Tremella Nostoc, Durr, Dr. on 397
Transdorf and Klaproth, their discoveries relative to the pheumalkali of Harhnemann 587
Trismus, or locked jaw, Bradley's case of 321
Triumph of medicine, Dr. Struve's review of 175
Trotter, Dr. on the Jennerian Inoculation
——— on cold affusion 75
——— on the concrete acid ib.
Ulcers, Edwards on 217
Urethra, Whately on strictures of the, reviewed 479
——— Cartwright on an improved mode of applying caustic to strictures in the 513
Uterus inverted, Dr. Welsh's case of 450
Vaccinia, White on 243
Vaccine inoculation, the Rev. Mr. Jenner on 401
Ventenat on the genus of lime tree 394
Vaccina, Philips on 548
Vaccine pock institution, removal of it 400
Vaccine inoculation, see *Inoculation vaccine* 384
——— Pearson, Dr. on 86
Vaccine, Boswell's case of 341
——— Ring on 354
——— Simmons on 134
——— Pole's case of 340
Vauquelin, his mode of extracting malat of lime from house leek
——— his discovery of silicious
Vaccine inoculation, Woodville, Dr. on 6
earth in antimony 199
——— his mode of preparing emetic tartar 199
Varus and Valgus, Watt on 119
Vaccine, Hutchinson on 359
Veins of horses, Prof. Abildgaard on injecting medicines into them 399
Vegetables, Hermbstaadt's chemical analysis of 361—467
Vinegar, ice, Prof. Lowitz's new mode of preparing 395
Wagstaff, his case of inflamed mamma 239
Walter, Prof. review of his book on

Page.
diseases of the kidnies and bladder 284
Water cold in dysentery, Brefeld on the use of 141
Watt on varus and valgus 199
——— Sheldrake in reply to 264
——— on distortions of the feet 426
——— on vaccine inoculation 430
Wantzel, Dr. his observations on Mr. Herne's account of the retina of the eye 585
Westminster hospital, see *diseases in the Westminster hospital*
Welsh, Dr. his case of inverted uterus 450
Westminster Hospital, diseases in, v. *Diseases in the Westminster Hospital* 16
Whyte on vaccinia 243
——— on the plague 245
Whately, Mr. on the gonorrhea virulenta in men, review of 379
——— Mr. on strictures in the Urethra, review of 479
White swelling, Lambs case of 346
Willan, Dr. Review of his reports on the diseases in London 482
Wichman's, Dr. his theory of difficult dentition reviewed.
Wood, rotten, on the illumination of 853
Woodville, Dr. on vaccine inoculation 6
Women, pregnant, Dr. Lehnhardt's salubrious liquor for 399
Yeast, Custance on 144
——— Bradley, Dr. on 145
——— Grore on, in reply to Custance
——— Edlin on 422
Yelloley on the radial artery 282
Yellow fever, in Philadelphia, Dr. Rush on, reviewed 291
Yeast, Rolfe on the use of in typhus and and putrid fever 508

REFERENCES TO THE PLATES.

Mr. Simmon's case of an extraordinary enlargement of the clitoris 1
Mr. Sheldrape's plates representing distorted feet 12
Mr. Spry's case hernia of the stomach, through the tendinous orifice of the diaphragm to the felis catus domesticus 105
Mr. Bailey's case of exfoliation 497

